# Complete Genome Sequence of Streptomyces albus Strain G153

**DOI:** 10.1128/mra.00332-22

**Published:** 2022-06-02

**Authors:** Tomoki Takeda, Nodoka Fukumitsu, Satoshi Yuzawa, Kazuharu Arakawa

**Affiliations:** a Institute for Advanced Biosciences, Keio University, Tsuruoka, Yamagata, Japan; b Systems Biology Program, Graduate School of Media and Governance, Keio University, Fujisawa, Kanagawa, Japan; c Department of Biotechnology, Graduate School of Agricultural and Life Sciences, The University of Tokyo, Tokyo, Japan; d Biological Systems and Engineering Division, Lawrence Berkeley National Laboratory, Berkeley, California, USA; e Faculty of Environment and Information Studies, Keio University, Fujisawa, Kanagawa, Japan; University of Maryland School of Medicine

## Abstract

The genus *Streptomyces* is a promising source of biologically active secondary metabolites. Here, we report the complete genome sequence of Streptomyces albus strain G153. The assembled genome comprised a single linear chromosome of 6.9 Mbp with a G+C content of 73.3%.

## ANNOUNCEMENT

Several Streptomyces albus and closely related strains are used as heterologous hosts for diverse secondary metabolite production (1, 2). Among them, Streptomyces albidoflavus J1074 (formerly known as Streptomyces albus J1074) is one of the most popular host strains, for which the genome sequence is available (3). However, the genome sequence of *S. albus* G153 has not yet been determined and a difference between them still remains elusive. Here we report a complete genome sequence for *S. albus* G153.

S. *albus* G153 was obtained from Tomohisa Kuzuyama cultured under aerobic conditions at 30°C for 3 days (100 mL of TSB medium [Oxoid] containing 50 mg/L of nalidixic acid [Nacalai] in a 300-mL baffled flask). Approximately 1.0 × 10^9 cells were collected and the genomic DNA was purified using Genomic-tips 20/G (Qiagen). Long read sequencing libraries were prepared and multiplexed using the Rapid Barcoding Kit (SQK-RBK004; Oxford Nanopore Technologies). Libraries were sequenced in a FLO-MIN106 flowcell, basecalled (guppy version 5.0.12, Super-Accurate Mode), demultiplexed and adapter-trimmed on the GridION X5 device (GridION software release 21.05.25, Oxford Nanopore Technologies). Long reads were quality checked using Nanoplot version 1.20.0 (4), which totaled 495,782,602 bp consisting of 125,903 reads of N50 length 8,787 bp. Reads longer than 5 kb (approximately x50 coverage) were used for assembly using Canu version 2.2 (5). The resulting single contig was manually confirmed to be full-length linear chromosome like other *Streptomyces* genomes by comparing it with the J1074 genome. A library for Illumina sequencing for error correction was prepared using a KAPA HyperPlus kit (Kapa Biosystems), and the library was sequenced on a NextSeq 500 sequencer (Illumina) using the 75-cycle high-output mode as single ends. Unfiltered 25,694,300 (1.9 Gbp) Illumina short reads were used for error correction with one round of Pilon version 1.2.4 (6). The assembly quality was assessed using Benchmarking Universal Single-Copy Orthologs (BUSCO) v.1 on the gVolante server (7), and the completeness score reached 100%. The genome was annotated using the DDBJ Fast Annotation and Submission Tool (DFAST) version 1.4.0 (8). All software was used with default settings unless otherwise specified.

The annotated linear genome of S. *albus* G153 is 6,850,711 bp with a G+C content of 73.3%, containing 6,072 putative coding sequences (CDSs), 21 rRNA genes, 77 tRNA genes, and five CRISPR loci were predicted. D-GENIES (9) comparison with S. *albus* J1074 revealed only 0.04% mismatched regions, and the Mauve version 2.4.0 (10) alignment revealed an 11,997-bp long insertion sequence (3,292,629 to 3,304,629 bp) in the G153 genome, in which a total of six CDSs were coded, including those annotated as LuxR family transcriptional regulators. LuxR family proteins are often involved in the quorum sensing mechanisms (11) and activate biosynthetic gene clusters in *Streptomyces* strains (12).

### Data availability.

The genome sequences reported here were deposited in DDBJ under accession numbers AP025687, and the raw reads were deposited in the Sequence Read Archive (SRA) under BioProject accession number PRJNA820546 as SRR18498194 and SRR18498195 runs.

## References

[1] Bu QT, Li YP, Xie H, Li JF, Lv ZY, Su YT, Li YQ. 2021. Rational engineering strategies for achieving high-yield, high-quality and high-stability of natural product production in actinomycetes. Metab Eng 67:198–215. doi:10.1016/j.ymben.2021.06.003. 34166765

[2] Liu Z, Zhao Y, Huang C, Luo Y. 2021. Recent advances in silent gene cluster activation in streptomyces. Front Bioeng Biotechnol 9:632230. doi:10.3389/fbioe.2021.632230. 33681170 PMC7930741

[3] Zaburannyi N, Rabyk M, Ostash B, Fedorenko V, Luzhetskyy A. 2014. Insights into naturally minimised Streptomyces albus J1074 genome. BMC Genomics 15:97. doi:10.1186/1471-2164-15-97. 24495463 PMC3937824

[4] De Coster W, D'Hert S, Schultz DT, Cruts M, Van Broeckhoven C. 2018. NanoPack: visualizing and processing long-read sequencing data. Bioinformatics 34:2666–2669. doi:10.1093/bioinformatics/bty149. 29547981 PMC6061794

[5] Koren S, Walenz BP, Berlin K, Miller JR, Bergman NH, Phillippy AM. 2017. Canu: scalable and accurate long-read assembly via adaptive k-mer weighting and repeat separation. Genome Res 27:722–736. doi:10.1101/gr.215087.116. 28298431 PMC5411767

[6] Walker BJ, Abeel T, Shea T, Priest M, Abouelliel A, Sakthikumar S, Cuomo CA, Zeng Q, Wortman J, Young SK, Earl AM. 2014. Pilon: an integrated tool for comprehensive microbial variant detection and genome assembly improvement. PLoS One 9:e112963. doi:10.1371/journal.pone.0112963. 25409509 PMC4237348

[7] Nishimura O, Hara Y, Kuraku S. 2017. gVolante for standardizing completeness assessment of genome and transcriptome assemblies. Bioinformatics 33:3635–3637. doi:10.1093/bioinformatics/btx445. 29036533 PMC5870689

[8] Tanizawa Y, Fujisawa T, Nakamura Y. 2018. DFAST: a flexible prokaryotic genome annotation pipeline for faster genome publication. Bioinformatics 34:1037–1039. doi:10.1093/bioinformatics/btx713. 29106469 PMC5860143

[9] Cabanettes F, Klopp C. 2018. D-GENIES: dot plot large genomes in an interactive, efficient and simple way. PeerJ 6:e4958. doi:10.7717/peerj.4958. 29888139 PMC5991294

[10] Darling AC, Mau B, Blattner FR, Perna NT. 2004. Mauve: multiple alignment of conserved genomic sequence with rearrangements. Genome Res 14:1394–1403. doi:10.1101/gr.2289704. 15231754 PMC442156

[11] Zhang W, Li C. 2015. Exploiting quorum sensing interfering strategies in gram-negative bacteria for the enhancement of environmental applications. Front Microbiol 6:1535. doi:10.3389/fmicb.2015.01535. 26779175 PMC4705238

[12] Shi Y, Gu R, Li Y, Wang X, Ren W, Li X, Wang L, Xie Y, Hong B. 2019. Exploring novel herbicidin analogues by transcriptional regulator overexpression and MS/MS molecular networking. Microb Cell Fact 18:175. doi:10.1186/s12934-019-1225-7. 31615513 PMC6794829

